# *In vivo* evaluation of an adaptive resuscitation controller using whole blood and crystalloid infusates for hemorrhagic shock

**DOI:** 10.3389/fbioe.2024.1420330

**Published:** 2024-11-08

**Authors:** Eric J. Snider, Saul J. Vega, I. Amy Nessen, Sofia I. Hernandez Torres, Sophia Salazar, David Berard, Jose Salinas

**Affiliations:** Organ Support and Automation Technologies Group, U.S. Army Institute of Surgical Research, San Antonio, TX, United States

**Keywords:** controller, hemorrhage, shock, animal testing, control systems, physiological closed-loop controllers, fluid resuscitation, automation

## Abstract

**Introduction:**

Hemorrhage remains the leading cause of preventable death on the battlefield. The most effective means to increase survivability is early hemorrhage control and fluid resuscitation. Unfortunately, fluid resuscitation requires constant adjustments to ensure casualty is properly managed, which is often not feasible in the pre-hospital setting. In this study, we showed how an adaptive closed-loop controller for hemorrhage resuscitation can be used to automate hemodynamic management using a swine hemorrhagic shock injury model.

**Methods:**

The adaptive resuscitation controller (ARC) was previously developed to track pressure–volume responsiveness in real time and adjust its infusion rate to reach the target mean arterial pressure (MAP). Swine while maintained under a surgical plane of anesthesia and analgesia underwent a splenectomy, followed by two hemorrhage and resuscitation events. For the first resuscitation event, hemorrhage was induced to reduce the MAP to 35 mmHg until arterial lactate reached 4 mmol/L. The ARC system then infused whole blood (WB) to reach the target MAP and maintained the subject using crystalloids for 120 min. For the second resuscitation event, the subjects were hemorrhaged again but resuscitated using only crystalloid infusion to reach the target MAP and 120-min maintenance.

**Results:**

The ARC was effective at WB resuscitation, reaching the target MAP in 2.0 ± 1.0 min. The median performance error was 1.1% ± 4.6%, and target overshoot was 14.4% ± 7.0% of the target MAP. The ARC maintained all animals throughout the 120 min maintenance period. For the second crystalloid-based resuscitation, ARC required a longer time to reach the target MAP, at an average rise time of 4.3 ± 4.0 min. However, target overshoot was reduced to 8.4% ± 7.3% of the target MAP. Much higher flow rates were required to maintain the target MAP during the second resuscitation event than during the first resuscitation event.

**Discussion:**

The ARC was able to rapidly reach and maintain the target MAP effectively. However, this sometimes required large volumes of fluid as the ARC’s only goal was to reach the target MAP. Further clinical insight is needed regarding the preferred aggression level to achieve the target MAP. In conclusion, the ARC was successful in its programmed objective of reaching and maintaining the target MAP for extended periods of time *in vivo*, a critical next step toward improving hemorrhage treatment in the pre-hospital environment.

## Introduction

Studies have shown that trauma is the third leading cause of death in the United States, second only to cancer and heart disease (Ahmad et al., 2023). Among military and civilian trauma patients, hemorrhage is the leading cause of potentially preventable mortality after injury. For civilian environments, an analysis of trauma-related deaths in a single large county in Texas during 2014 found that 44.9% of all hemorrhage-related deaths could be considered preventable, had optimal care been available (Drake et al., 2020). On the battlefield, an analysis of combat casualties in Iraq and Afghanistan between 2001 and 2011 found that 90.9% of potentially preventable deaths occurred due to hemorrhage (Eastridge et al., 2012). Death from hemorrhagic shock is caused by the loss of circulating blood volume that results in the disruption of oxygen delivery, leading to a lack of oxygen perfusion in vital organs and, ultimately, to cell and tissue death (Hinojosa-Laborde et al., 2022). Mortality due to hemorrhage can be prevented by early hemorrhage control, access to definitive surgical care, and restoration of the circulatory volume via fluid infusion, with whole blood (WB) being the resuscitation fluid of choice (Cap et al., 2018a).

In resource-austere environments, such as far-forward military units and mass casualty incidents, as well as in rural and wilderness settings, limited access to skilled care providers may reduce the probability of survival for hemorrhaging patients. This access to providers will likely worsen on the future battlefield, where a contested airspace will limit evacuation opportunities, resulting in prolonged field care for up to 72 h (Townsend and Lasher, 2018). Extended prolonged field care will require continuous fluid monitoring to ensure patient stability through worsening injuries or untreated internal injuries, leading to re-bleed events that must be stabilized. In these environments, the use of automated technologies or physiological closed-loop controllers (PCLCs) that require little to no human intervention to deliver the necessary management of trauma patients will improve the care of the patients and survivability rates (Snider et al., 2022c).

Automated control is particularly useful when complex systems need to be controlled with speed, accuracy, and precision that exceed human capabilities or when the amount and/or duration of control make it impractical for a human operator alone to attend to the system. Satellite dish positioning (Li et al., 2017), large-scale manufacturing operations (Rupenyan and Balta, 2024), and missile guidance systems (Tsourdos and White, 2001) are examples of the former, while climate control systems (Ala’raj et al., 2022) and water quality monitoring (Bórquez López et al., 2020) are examples of the latter. Because of the relatively slow dynamics and varying nature of hemodynamics, automated fluid resuscitation fits more in the second group, although a properly tuned controller will generally provide accuracy and stability benefits above strictly manual control. Automated control can be broadly divided into two groups: provider-in-loop and closed-loop systems. Provider-in-loop systems are only semi-automated, where the controller may run on its own, but the user ultimately regulates the controller’s output. Closed-loop control systems are near, if not fully, automated. Although certain initial conditions and target parameters may be input by the user, once the closed-loop controller is activated, all its outputs are calculated and executed by the controller itself until the system is stopped. There are also “hybrid” controllers that are unique, where they primarily function as closed-loop but make allowances for the user to manually override output values or change targets without having to stop and reset the controller (Avital et al., 2022).

Previously, we developed an adaptive resuscitation controller (ARC) using *in vitro* methods to develop and optimize its design for hemorrhagic shock resuscitation (Berard et al., 2022; Snider et al., 2022b; Vega et al., 2023). An important next step toward the deployment of this controller is the use of this system to manage hemorrhagic shock in animal models. In this study, the ARC’s performance for managing resuscitation in a large animal model of life-threatening hemorrhage was evaluated. The primary outcome was to evaluate the ARC’s performance for whole blood (WB) resuscitation, while the secondary outcome for this effort was to similarly evaluate the ARC’s performance for crystalloid-based resuscitation during a re-bleed event.

A number of other PCLCs have been developed and are actively in development in addition to the ARC. We previously performed a scoping review of these technologies for fluid resuscitation (Avital et al., 2022) and compared the ARC’s performance against a wide range of conventional controller designs using a hardware-in-loop test platform (Snider et al., 2022b). Comparisons to proportional–integral–derivative (PID), fuzzy-logic, and decision table approaches highlighted some of the benefits of the ARC design, where it can adapt its flow rate more readily to the subject to overcome changes in fluid responsiveness or resuscitate through active hemorrhage situations. Although the ARC outperformed traditional controller designs in these studies, this present effort was its first use in a live animal study, in contrast to the ideal hardware-in-loop model it was developed using. As for other PCLC technology in development, most other resuscitative PCLCs have been designed for routine surgical support, which is generally a less intensive environment for hemorrhage control (Kramer et al., 2008; Rinehart et al., 2011; 2015; Bighamian et al., 2016; Libert et al., 2021). In addition, most of these situations and, thus, the scope for these other controller designs did not include WB as the infusate. Compared to other fluid types, WB has very different pressure–volume dynamics that were explicitly considered in the ARC’s design.

## Materials and methods

### Overview of the adaptive resuscitation controller

**FIGURE 1:**
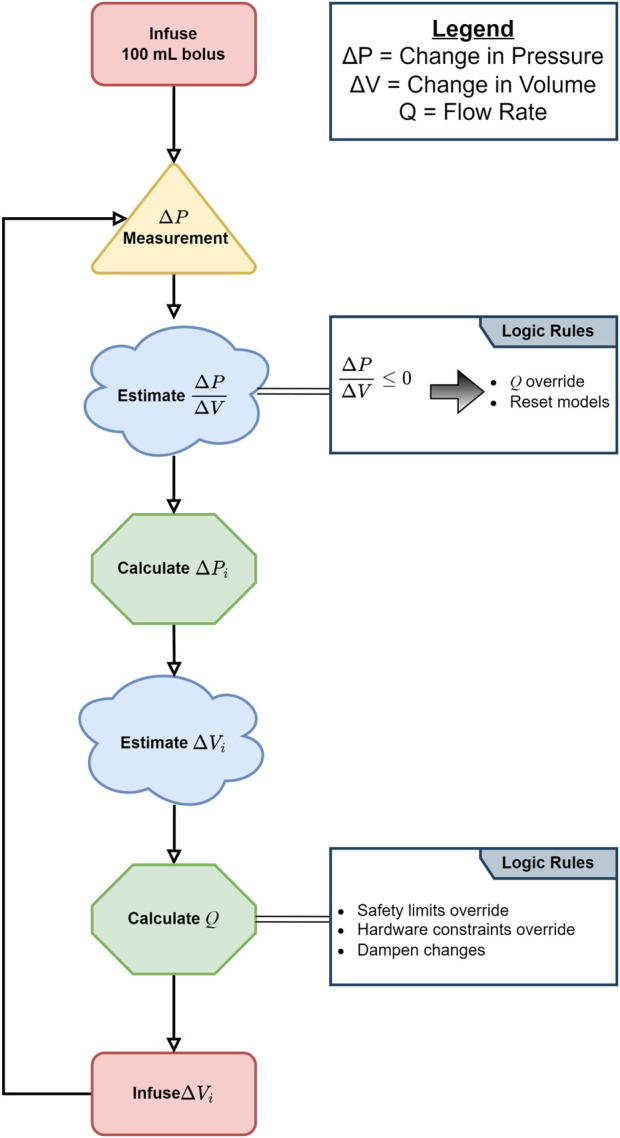
Diagram of the adaptive resuscitation controller (ARC) logic.

In previous work, we developed an adaptive resuscitation controller for the treatment of hemorrhagic shock that continuously assesses a subject’s responsiveness to fluid infusion as a way of estimating an appropriate infusion rate (Vega et al., 2023). As shown in Figure 1, the ARC measures the change in the mean arterial pressure (MAP) (Δ*P*) that occurs as a consequence of infusing a known volume of fluid (Δ*V*) over a relatively short period of time (Δ*T*) and then uses a set of regression models to estimate the additional volume needed to achieve the desired target pressure. This controller was tuned to achieve a desired resuscitation pace (Δ*P*/Δ*T*), which is then used to calculate the infusion flow rate (Q). Tuning was done using datasets obtained from previous animal studies, where swine underwent an uncontrolled hemorrhage injury (Sondeen et al., 2007), and its performance was ultimately evaluated on a hardware-in-loop test platform (Snider et al., 2022b). These tuning experiments resulted in an optimal resuscitation pace of 6 mmHg/min.

The ARC was implemented in MATLAB (MathWorks, Natick, MA, United States) running on a standard personal computer, which was connected to an Infinity Delta XL patient monitor (Dräger Medical, Lübeck, Germany) via a PowerLab data-acquisition unit (ADInstruments, Dunedin, New Zealand). The same computer was also connected via a standard serial link to a Masterflex L/S peristaltic pump (Cole-Parmer, Vernon Hills, IL, United States). In this configuration, the ARC would receive continuous arterial pressure measurements from the patient monitor and send commands to the pump to infuse fluids into the hemorrhaged subject. For the present *in vivo* study, the same software and hardware systems were used, but a few additional modifications were made as described in the sections below.

### Hemorrhagic shock animal study for ARC evaluation

#### Overview of animal preparation and instrumentation

Research was conducted in compliance with the Animal Welfare Act, implementing Animal Welfare regulations and the principles of the Guide for the Care and Use for Laboratory Animals. This study was approved by the Institutional Animal Care and Use Committee (IACUC) at the United States Army Institute of Surgical Research. The facility where this research was conducted is fully accredited by the AAALAC International. All study manipulations were performed in accordance with IACUC-approved protocol A-23-006.

**FIGURE 2:**
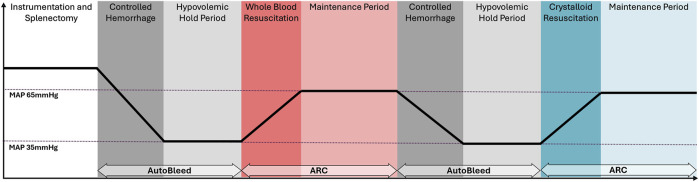
Timeline for the hemorrhagic shock *in vivo* study for the evaluation of the adaptive resuscitation controller (ARC).

A swine (*Sus scrofa domestica*) hemorrhagic shock and resuscitation model was used to validate the ARC in an open-label, prospective, non-randomized, single-group pilot study. The study utilized n = 15 approximately 4-month-old, intact female Yorkshire swine weighing approximately 40 kg at the time of study. The model consisted of two controlled hemorrhages in the animal until they reached a state of hypovolemic shock defined as a MAP of 35 mmHg with an arterial blood lactate level of 4 mmol/L or higher, each followed by an ARC-managed fluid resuscitation—the first resuscitation was done using WB and the second with lactated Ringer’s (crystalloid) solution (Figure 2). The animals were given analgesia with buprenorphine SR (Wedgewood Pharmacy; Swedesboro, NJ) and maintained under a surgical plane of anesthesia throughout the study with either inhalation of isoflurane or total intravenous anesthesia (TIVA) using ketamine and midazolam or propofol.

First, the swine were instrumented with catheter insertion to four vessels: arterial line (Arrow International, Morrisville, NC, United States) in the carotid for pressure readings; femoral arterial line for controlled hemorrhage and blood sampling; femoral venous line (Arrow International, Morrisville, NC, United States) for resuscitation; and a percutaneous sheath introducer for the insertion of a pulmonary artery Swan-Ganz catheter (Edwards Lifesciences, Irvine, CA, United States) for continuous cardiac output monitoring.

After all lines were placed and subjects were allowed to stabilize for 20 min, an open splenectomy procedure was performed, during which epinephrine was applied to vasoconstrict the spleen (Bakovic et al., 2013). The spleen was removed after vascular ligation using surgical clips (Weck^®^ Hem-o-lok^®^, Teleflex Medical, Wayne, PA, United States). After closing the incision, the subject was turned to the prone position and allowed to stabilize for a 30-min period. During this period, the anesthetic regimen was switched to TIVA with ketamine (0–10 mg/kg/hr) and midazolam (0–2 mg/kg/hr) (n = 8). The original intent for the study was to switch to ketamine for all subjects, but due to a nationwide shortage of this drug at the time, this was not possible. Instead, one subject was transitioned to propofol (0–18 mg/kg/hr) (n = 1), and the remainder continued with isoflurane (n = 6) anesthesia throughout the study.

### First hemorrhage event

After the stabilization period, a controlled arterial hemorrhage was initiated to a target MAP using an automated hemorrhage decision table (AutoBleed). AutoBleed draws blood from the subject using a peristaltic pump until it reaches a user-set MAP target and maintains the pressure at this level by continuously drawing more blood or re-infusing it back, as needed. Rates are simply set by AutoBleed and not adaptive, instead using rules that decide rates based on the distance from the target MAP. When withdrawing blood, AutoBleed also controls a second peristaltic pump to mix CPDA-1 (citrate, phosphate, dextrose, and adenine) solution at a volumetric ratio of 1:7 with the withdrawn blood, which is then stored in a sterile blood bag. For this study, AutoBleed was implemented in MATLAB and set to reach a target MAP of 35 ± 2 mmHg indefinitely until manually stopped by an operator. AutoBleed was only active during the hemorrhage and shock phases of the study and was disengaged during resuscitation.

The WB withdrawn during hemorrhage was collected in blood bags and kept in a custom-fitted holder inside a shaking incubator set at 37°C. The incubator equipment was placed on top of a scale to keep track of the weight of total blood loss. When the MAP target of 35 mmHg was met, the subject was kept in the hypovolemic state until lactate levels reached or surpassed 4 mmol/L or for a total of 90 min, whichever happened first. Lactate readings were measured every 10 min during this period by obtaining a 0.5-mL arterial blood sample and analyzing it using an iSTAT CG4^+^ Cartridge (Abbott Point of Care, Princeton, NJ, United States).

### ARC-based resuscitation

Once a shock criterion was met, AutoBleed was disengaged, CaCl_2_ (1 g/10 mL bolus) was injected into the subject, and resuscitation controlled by the ARC was initiated to re-infuse the autologous blood collected during the controlled hemorrhage until a target MAP of 65 ± 5 mmHg was reached. A target MAP of 65 mmHg was chosen based on damage control resuscitation recommendations to maintain permissive hypotension during trauma resuscitation (Cap et al., 2018b; Leibner et al., 2020). Joint Trauma System Guidelines of a systolic blood pressure target of 100 mmHg were estimated as a target MAP of 65 mmHg for this study (Damage Control Resuscitation - Joint Trauma System, 2024).

Occasionally, a MAP target of 65 mmHg could not be reached despite fluid infusion, presumably due to anesthetic-induced vasodilation. To overcome challenges where reaching the target MAP was not feasible without significant fluid overload, a central venous pressure (CVP) threshold of 15 mmHg was set. If the CVP surpassed this threshold, the target MAP was first reduced to baseline MAP for the subject, followed by iterative reductions of 2 mmHg until a stable MAP was reached, if necessary. Once the target pressure was reached, a 10-min stabilization period allowed the controller and pressures to reach equilibrium, and the resuscitation fluid was transitioned to crystalliod for a 120-min-long maintenance period. Given that whole blood is a scarce resource in both civilian and military settings, we performed an early switch to lactated Ringer’s solution to replicate likely resource constraints that the ARC may be faced with. During the maintenance period, arterial blood lactate samples were collected following the same procedure as for the hypovolemic hold samples, with the first sample collected right after the 10-min stabilization period and then every 60 min subsequently.

### Second hemorrhage event and resuscitation

Upon completion of the first maintenance period, a second AutoBleed-controlled hemorrhage event was initiated to the same hypovolemic target of 35 ± 5 mmHg. This second hemorrhage was performed to evaluate the ARC’s performance with a different infusate, as well as to examine the performance during re-bleed events that will be more prevalent in a prolonged field care setting. The second hypovolemic hold period was then set to simply match the duration of the first hypovolemic hold. However, to minimize the blood lactate buildup during the second hemorrhage event, an additional criterion was added to shorten the hold period if lactate levels reached or surpassed 6 mmol/L. This hypovolemic phase was followed by a second resuscitation by the ARC using crystalloid as the infusate until the MAP target of 65 ± 5 mmHg was reached, repeating 10-min stabilization, followed by a 120-min maintenance period. Again, if the CVP reached or surpassed 15 mmHg, the target MAP was reduced by 2 mmHg until a stable pressure was reached. Following the maintenance period for the second resuscitation, all study events ended, and the subject was humanely euthanized with an overdose of sodium pentobarbital (FatalPlus, Vortech; Dearborn, MI).

### Controller and data collection setup

During the experiments, the control system operator would start either the AutoBleed or ARC controller at the time points prescribed by the study protocol. Changes to the infusion fluid type were performed manually by the system operator, as dictated by the protocol. Throughout the study, continuous arterial and venous pressures, along with traditional vital signals and waveforms, were captured from the patient monitor. When a Swan-Ganz catheter was placed in the subjects, a HemoSphere monitoring device (Edwards Lifesciences) was used for capturing cardiac output (CO) and mixed venous saturation (SvO_2_). Analog and digital data were captured on a purpose-built data acquisition system at a sampling rate of 500 Hz for analog waveforms and 1/5 Hz for all other digital recordings. Weight scale readings for capturing the hemorrhage volume of the blood bags were also recorded by this system, as well as flow rates for both peristaltic pumps.

### Data processing and performance metrics

The arterial pressure data for each animal were analyzed independently, and the average mean arterial pressure was calculated for all subjects. Additional averaged datasets were produced for CVP, SvO_2_, CO, and stroke volume variability (SVV) to identify unique physiological trends that may not be captured by the arterial pressure alone. Blood analysis results, such as blood lactate levels and hematocrit, were similarly processed for each subject across the experimental phases. A selection of commonly used controller metrics was used to obtain objective scores for the ARC’s performance at reaching and maintaining the target pressure (Varvel et al., 1992; Marques et al., 2017; Mirinejad et al., 2020). As these metrics have been described previously (Vega et al., 2023), a list of the metrics calculated is provided as follows: (i) median performance error (MDPE); (ii) median absolute performance error (MDAPE); (iii) target overshoot; (iv) effectiveness; (v) resuscitation effectiveness; (vi) wobble; (vii) rise time efficiency; (viii) area above the target MAP; (ix) area below the target MAP; (x) median infusion rate; (xi) mean infusion rate; and (xii) infusion rate variability.

A new metric was added, termed “resuscitation effectiveness.” This novel metric is a modification of the existing metric “effectiveness,” which is defined as the percentage of resuscitation time that pressure was held between 5 mmHg above or below the target MAP. Similar to the existing “effectiveness” metric, the novel “resuscitation effectiveness” metric was defined as the percentage of time the MAP was no more than 5 mmHg below the target MAP, but no penalty was associated with exceeding the target as it is less of a concern during hemorrhagic shock resuscitation. Performance metrics calculations and raw data processing were performed using Jupyter Notebook and Python. Final data analysis and visualization were performed using GraphPad Prism 10 (La Jolla, CA, United States).

## Results

### Overview of animal model performance

**FIGURE 3:**
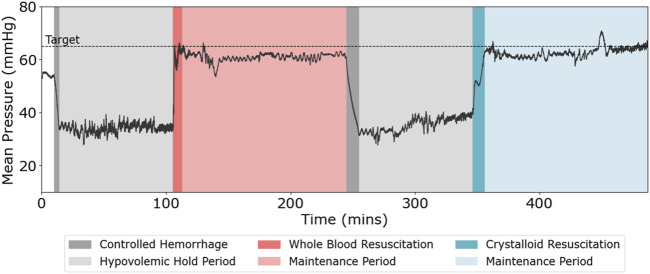
Representative mean arterial pressure (MAP, mmHg) vs. time (minutes) across both hemorrhage and resuscitation events for the swine injury model.

The developed animal model allowed for the evaluation of the ARC’s performance through two consecutive hemorrhage injuries and resuscitation treatments. A representative MAP trace for one subject across the entire study is shown in Figure 3. First, the subjects underwent controlled hemorrhage to reach the MAP target of 35 mmHg, which was held for 90 min or until the blood lactate level reached 4 mmol/L. On average, across all subjects, this goal was reached in 41.7 ± 30.4 min (n = 12). Then, the ARC resuscitated to the MAP target of 65 mmHg with WB, followed by maintaining the pressure close to the target MAP with crystalloids for 120 min. The controlled hemorrhage was repeated for an equal duration to the first hemorrhage duration, followed by resuscitation to the same target MAP and maintaining for 120 min with only crystalloids.

**FIGURE 4:**
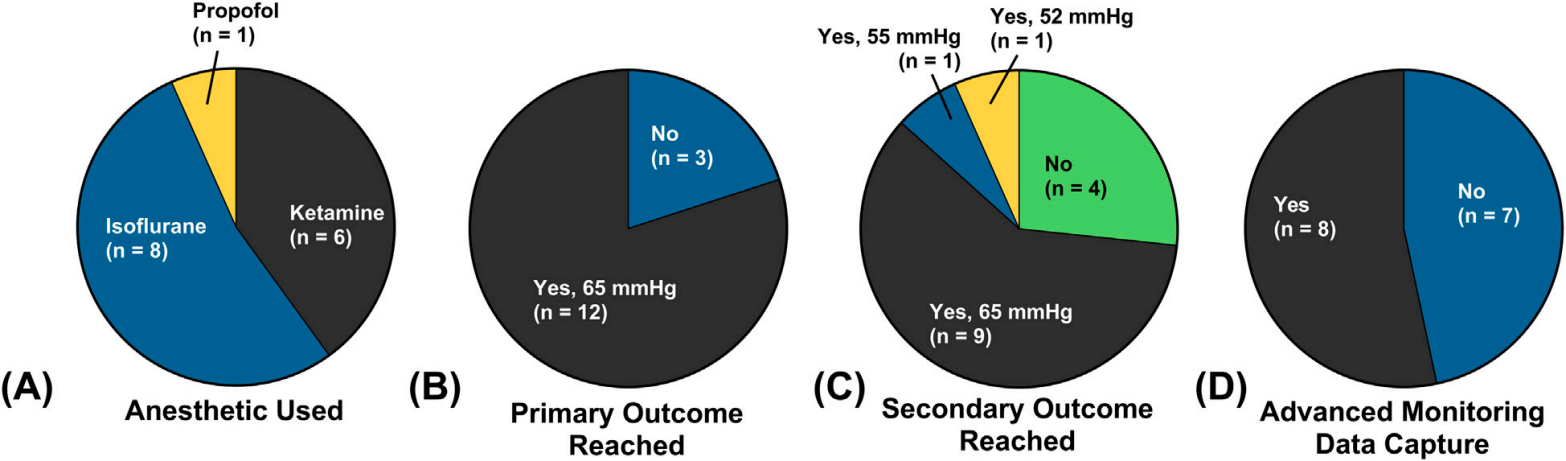
Summary of the animal study setup and outcomes based on **(A)** primary anesthetic agent used, **(B)** whether the primary outcome (survival through the first resuscitation event) was reached, **(C)** whether the secondary outcome (survival through the second resuscitation event) was reached, and **(D)** whether advanced monitoring via the Swan-Ganz catheter was collected.

A breakdown of outcomes and differences between the 15 swine used in this study is given in Figure 4. Of the 15 total swine used, 8 were maintained under a surgical plane of anesthesia and analgesia with isoflurane, 6 with ketamine as the primary anesthetic agent, and 1 with propofol (Figure 4A). Differences were due to nationwide ketamine shortages, limiting its use across the entire study. The primary study outcome, defined as successfully maintaining the target MAP for 120 min during the first resuscitation, was reached in 12 subjects (Figure 4B). The three swine that did not reach the primary outcome died due to ventricular fibrillation during the initial hemorrhage, which we believed to be due to complications with the Swan-Ganz catheter. Of the remaining 12 subjects, only 1 did not reach the secondary outcome due to having undergone a cardiac event during resuscitation with crystalloid. Another two subjects had their target MAP adjusted for the second resuscitation, based on elevated CVP. Lastly, CO and other advanced monitoring metrics were collected in a total of eight subjects due to complications with Swan-Ganz catheter placement in the others.

**FIGURE 5:**
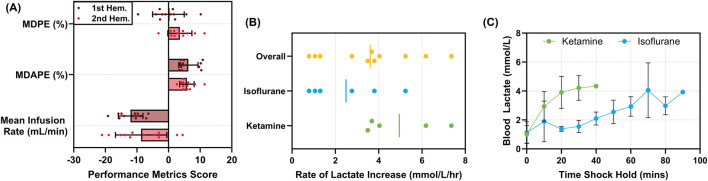
Characterization of the AutoBleed hypovolemic hold. **(A)** Median performance error (MDPE, %), median absolute performance error (MDAPE, %), and mean infusion rate (mL/min) for the first and second hemorrhage events (n = 12). **(B)** The rate of blood lactate increase (mmol/L/hr) during the first hypovolemic hold for all subjects (n = 11); data are shown for ketamine (n = 5) or isoflurane (n = 6) anesthetic. **(C)** Average blood lactate levels (mmol/L) vs. time (min) across the first hypovolemic hold for ketamine and isoflurane anesthetic. Data on the subject using propofol were excluded for **(B)** and **(C)**. Error bars denote standard deviation.

For the hypovolemic hold period managed by AutoBleed, the system reinfused WB at progressively quicker rates when the MAP drifted below 35 mmHg and withdrew more blood from the subject when the MAP increased above 35 mmHg. However, the average flow rates remained low across the hemorrhage hold window, with average rates being −12.0 ± 3.90 and −8.69 ± 8.13 mL/min for the first and second hemorrhage events, respectively (negative flow rate denoting fluid withdrawal; Figure 5A). The AutoBleed program maintained the pressure very close to the target MAP, with the MDPE at −0.061% ± 4.99% and 3.56% ± 3.81% of the target MAP for the first and second hemorrhage events, respectively (Figure 5A). The MDAPE was similar for both hemorrhage events, at 6.25% ± 3.03% and 5.78% ± 2.30%, respectively. Overall, the error percentages were low across both hemorrhages. By actively holding the subject at the hypotensive target MAP, the injury model attempted to overcome compensatory physiological mechanisms that may lessen the effects of the hemorrhage. On average, blood lactate levels increased at a rate of 3.61 ± 2.10 mmol/L/hr (Figure 5B). Differences in the rates of blood lactate levels were evident between the ketamine and isoflurane anesthetic used (Figure 5C). Overall, blood lactate accumulation rates were 4.95 ± 1.72 mmol/L/hr for ketamine and much slower for isoflurane anesthesia at 2.49 ± 1.77 mmol/L/hr, suggesting a potential hemorrhagic shock protective effect when using isoflurane (Figure 5B).

### Performance of the ARC during the first resuscitation event using whole blood

**FIGURE 6:**
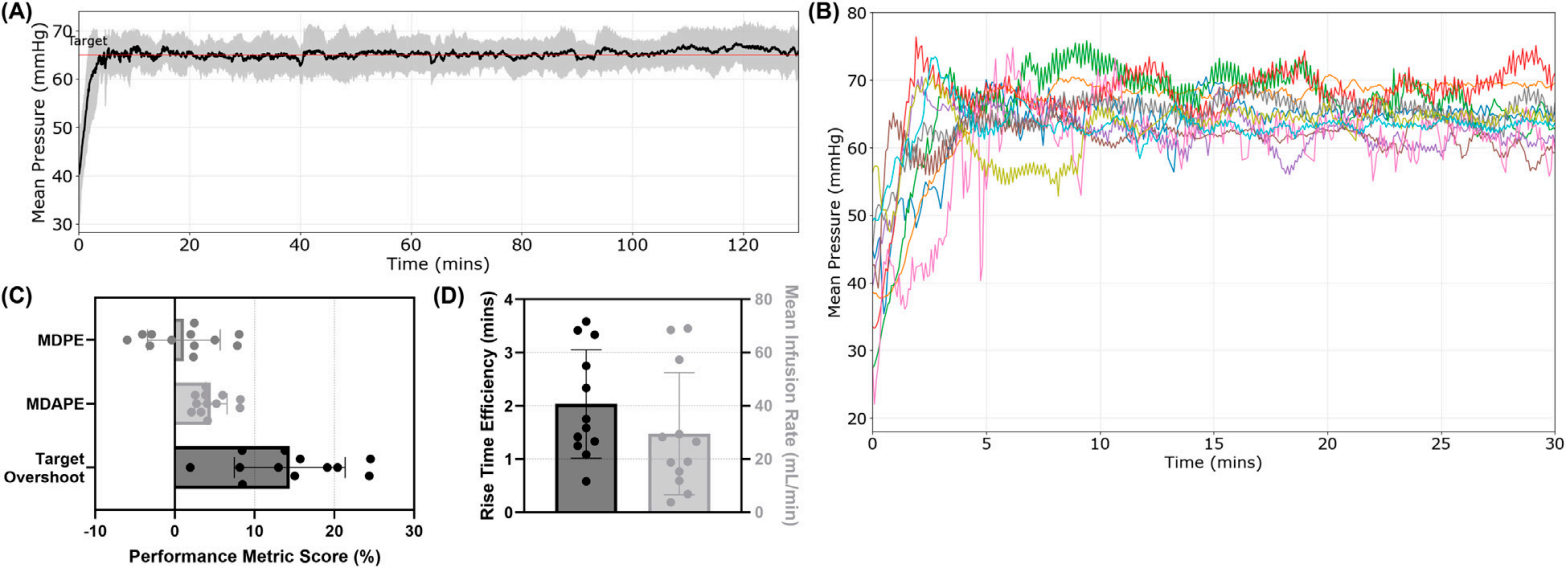
Summary of MAP trends during the first resuscitation controlled by the ARC. Whole-blood resuscitation was used to reach the target MAP, followed by a 120-min maintenance period using a crystalloid. **(A)** Average MAP trends vs. time (n = 12). Standard deviation is denoted by the shaded region, and the red line represents the target MAP. **(B)** MAP plots for each individual subject during the first resuscitation period. Performance for **(C)** MDPE, MDAPE, and target overshoot percentages of the target pressure and **(D)** rise time efficiency (minutes, left axis) and mean infusion rate (mL/min, right axis) are shown. Mean values are shown with error bars denoting standard deviation.

The first resuscitation utilized WB to resuscitate to a target MAP of 65 mmHg, followed by an additional 10 min of stabilization with WB as the infusate and then 120 min of crystalloid-based maintenance at the target MAP. On average, the ARC was successful in reaching and maintaining the target MAP for the entire 120-min window (Figure 6A). However, the approach to reach the target MAP was different for each subject based on pressure–volume responsiveness measured by the ARC, resulting in variability across the subjects (Figure 6B). On average, the MDPE was only 1.13% ± 4.55% of the target MAP, while the MDAPE was higher at 4.50% ± 2.03% (Figure 6C). The shift in error agreed with the target overshoot reaching as much as 14.4% ± 6.95% of the target MAP on average, likely due to the rapid rise time efficiency of 2.03 ± 1.02 min (Figure 6D). The overall average infusion rate across the first resuscitation event was 29.6 ± 22.9 mL/min (Figure 6D). The flow rate was adjusted constantly by the ARC, and total infused volumes varied greatly across each subject during the first resuscitation event (Supplementary Figure S1).

**FIGURE 7:**
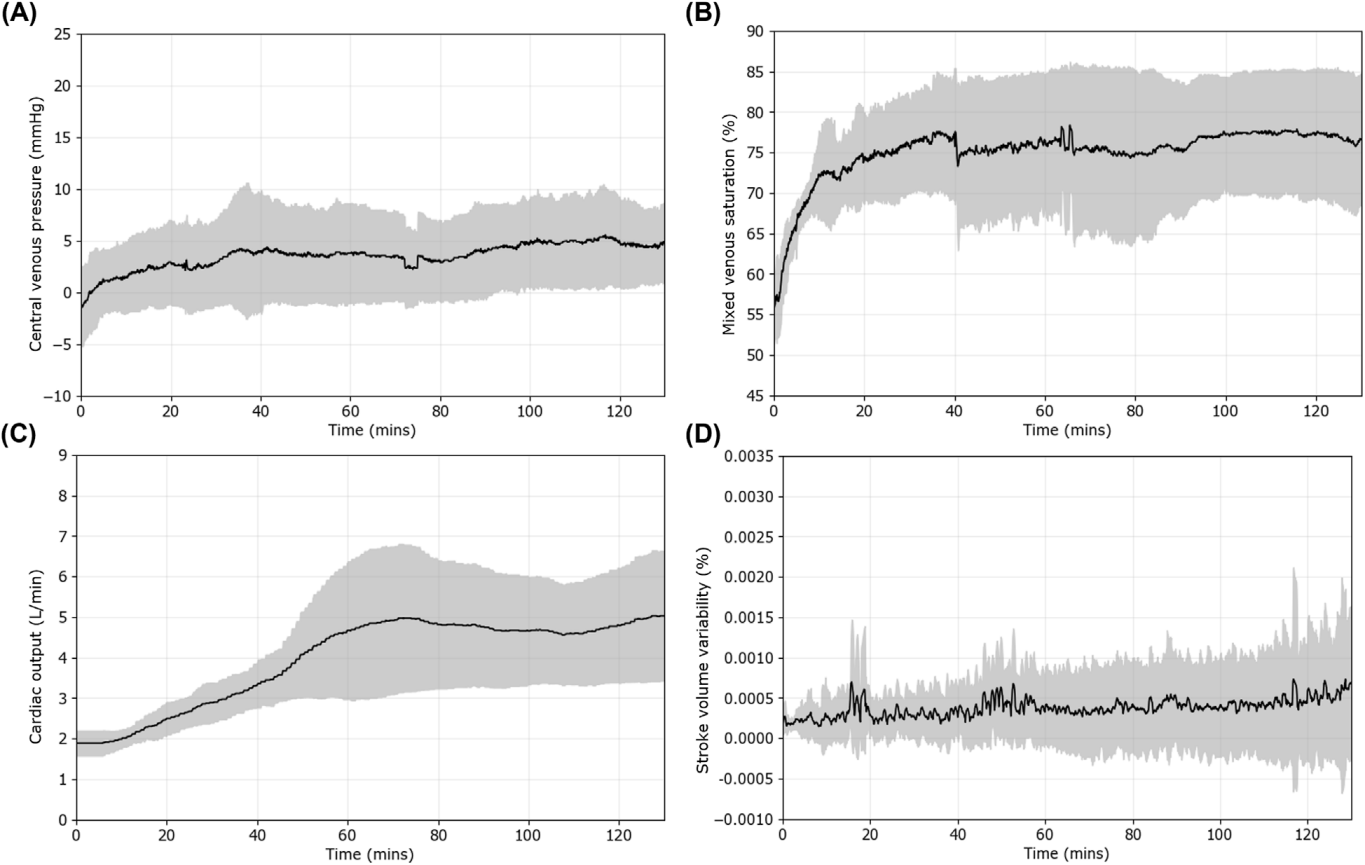
Additional vital sign trends during the first resuscitation period controlled by the ARC. Results are shown as the mean (n = 9 subjects), with shaded regions denoting standard deviation error bars for **(A)** central venous pressure (CVP), **(B)** mixed venous saturation (SvO_2_), **(C)** cardiac output (CO), and **(D)** stroke volume variability (SVV).

For other hemodynamic measurements, the CVP reached a negative value on average at the end of the hemorrhagic shock window (−0.9 ± 3.6 mmHg). It recovered to 0.8 ± 3.3 mmHg when the target MAP was reached and 5.3 ± 3.4 mmHg by the end of the maintenance period (Figure 7A). SvO_2_ decreased to 54.6% ± 3.9% at the start of resuscitation and quickly recovered to 62.9% ± 4.0% after reaching the target MAP (Figure 7B). CO took longer to recover, only reaching 1.91 ± 0.3 L/min after reaching the target but recovered to 5.22 ± 1.59 L/min by the end of the maintenance period (Figure 7C). We also looked at SVV as it has been shown to assess preload and fluid responsiveness (Figure 7D) (Hofer and Cannesson, 2011; Teboul et al., 2019). Overall, SVV slightly increased across the maintenance window, as did the variation within the SVV measurement (coefficient of variation of 79% at 0 min maintenance vs. 162% at 60 min vs. 130% at 120 min).

### Evaluation of ARC performance during the second resuscitation event with the crystalloid

**FIGURE 8:**
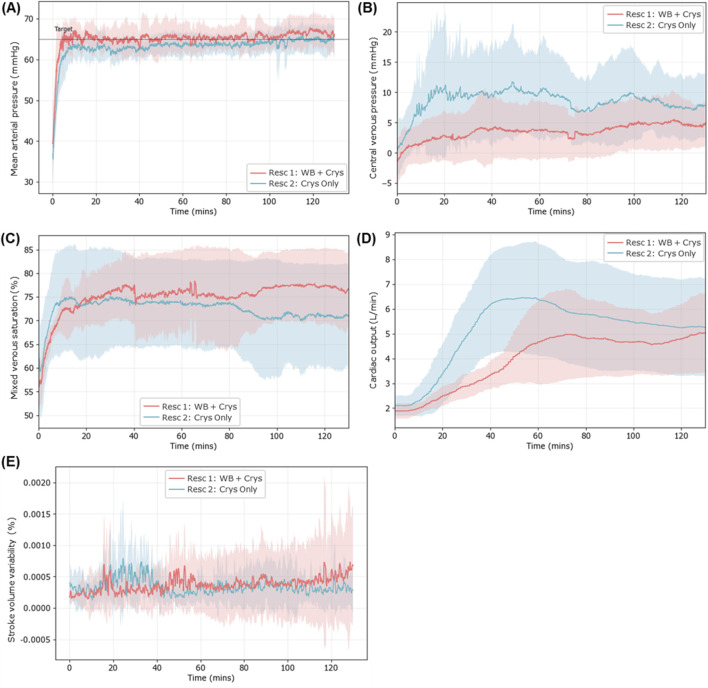
Physiological data trends during the second resuscitation event (blue), where the crystalloid infusate was used during the entire resuscitation and maintenance window to reach and maintain the target MAP. Results are compared against the first resuscitation event (red) for **(A)** average MAP trends (first resuscitation n = 12; second resuscitation n = 9), **(B)** central venous pressure (n = 8 for each), **(C)** mixed venous saturation (n = 8 for each), **(D)** cardiac output (n = 8 for each), and **(E)** stroke volume variability (n = 8 for each). Standard deviation is shown as the shaded region for both resuscitation events.

For the second resuscitation event, a crystalloid infusate was used both to reach the target MAP and maintain it for 120 min. Overall, the ARC was successful in reaching the target MAP and maintaining at pressure with the crystalloid during this second resuscitation (Figure 8A). Overall, the required fluid volume was higher during the second resuscitation event than during the first and varied significantly across each subject (Supplementary Figure S2). More differences were noted in other vital signs. The CVP reached much higher values during the second resuscitation than during the first, with values reaching 3.3 ± 3.9 mmHg after the target MAP was reached, 4.1 times higher than those in the first resuscitation event (Figure 8B). The difference between the two was reduced after 120 min (5.3 ± 3.4 mmHg first event vs. 7.9 ± 5.3 mmHg second event), but the difference was still 1.5 times higher on average.

Mixed venous saturation variability was higher immediately following resuscitation, with a coefficient of variation 6.4% vs. 12.3% in the first vs. second resuscitation event (Figure 8C). In the later stages, SvO_2_ decreased for the second resuscitation (70.9% ± 11%), while it did not in the first resuscitation event (76.6% ± 7.6%). CO peaked earlier and higher during the second resuscitation, reaching 5.84 ± 2.06 L/min after 60 min, while during the first resuscitation, it only reached 5.15 ± 1.76 L/min (Figure 8D). However, CO decreased during the later maintenance phase for the second resuscitation event while it continued to increase for the first resuscitation event. CO for both resuscitation events was at a similar point by the end of the maintenance period. SVV magnitudes remained similar with the exception of an elevation after 30 min for the second resuscitation that quickly dissipated. However, there were differences in the variability of SVV, which became more pronounced as it approached the end of the 120 min, with SVV standard deviation at only 0.00036% for the second resuscitation compared to 0.00086% for the first resuscitation (Figure 8E).

**FIGURE 9:**
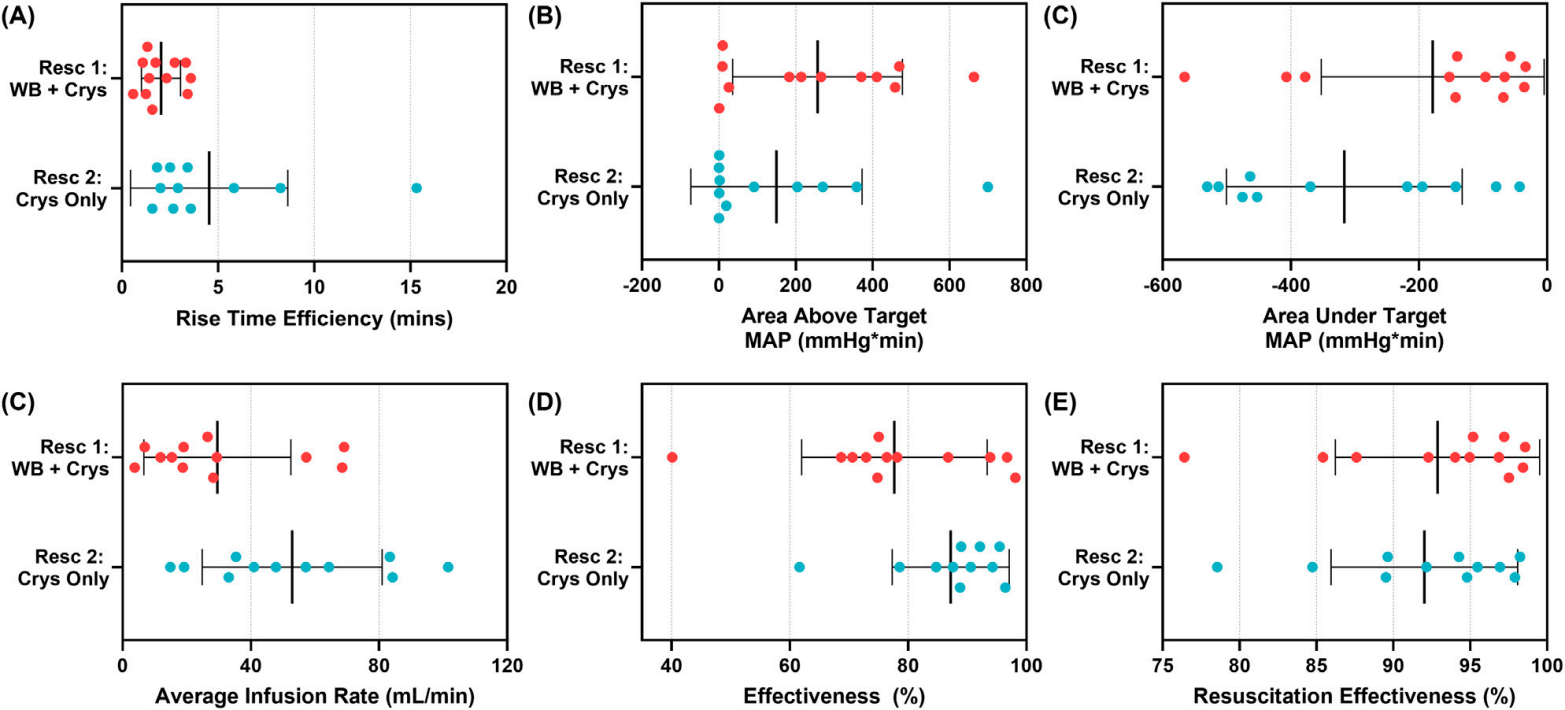
Comparison of selected performance metrics for first resuscitation (whole blood, n = 12) and second resuscitation (crystalloid, n = 11) using the ARC. **(A)** Rise time efficiency, **(B)** area above the target MAP and below the MAP response curve, **(C)** area under the target MAP and above the MAP response curve, **(D)** average infusion rate, **(E)** effectiveness, and **(F)** resuscitation effectiveness are shown as individual data points, with the mean result and standard deviation error bars shown.

In addition, conventional control system performance metrics were quantified to compare the ARC’s performance during the first and second resuscitation events. The ARC required more time to reach the target MAP during the second resuscitation, resulting in a higher rise time efficiency metric (2.03 ± 1.02 min first vs. 4.25 ± 4.02 min second resuscitation; Figure 9A). The slower rise time intuitively paired with the decreased area above the target MAP (256.7 ± 220.9 mmHg*min first vs. 138.6 ± 215.6 mmHg*min second resuscitation), due to reduced overshooting, and increased the area under the target MAP (−178.5 ± 173.9 mmHg*min first vs. −316.2 ± 175.4 mmHg*min second resuscitation; Figures 9B, C). Although it took longer to reach the target MAP, higher infusion rates were observed for the second resuscitation (51.7 ± 27.1 mL/min) than for the first resuscitation (29.6 ± 22.9 mL/min, Figure 9D). Overall, the effectiveness was higher for the second resuscitation than for the first resuscitation (Figure 9E). However, this was due to the slight target overshooting, which was more pronounced during the first resuscitation due to the pressure drifting too high. As target overshoot is less of a concern for hemorrhagic shock patients compared to target undershoot, we used a new metric, termed resuscitation effectiveness, that only penalized the ARC when the pressure was more than 5 mmHg below the target MAP. In doing so, resuscitation effectiveness was similar across both resuscitation events (92.9% ± 6.64% first vs. 92.1% ± 5.8% second resuscitation; Figure 9E). A summary of all performance metrics, standard deviations, and ratios between both resuscitation events is shown in Table 1.

**TABLE 1:** Summary of controller performance metrics for the first resuscitation event (n = 12) using whole blood and the second resuscitation event (n = 11) using crystalloid controlled by the ARC.

	Resuscitation 1: whole blood + crystalloid		Resuscitation 2: crystalloid only		Ratio resuscitation 1:2
	Mean	Standard deviation	Mean	Standard deviation	
MDPE (%)	1.13	4.55	−1.48	4.02	↓ −0.76
MDAPE (%)	4.50	2.03	4.25	1.28	↗ 1.06
Wobble (%)	1.45	0.51	1.36	0.44	↗ 1.06
Effectiveness (%)	77.68	15.70	87.67	9.58	↘ 0.89
Resuscitation effectiveness (%)	92.88	6.64	92.14	5.80	↗ 1.01
Target overshoot (%)	14.42	6.95	8.35	7.29	↑ 1.73
Area above the target MAP (mmHg*min)	256.73	220.87	138.61	215.63	↑ 1.85
Area below the target MAP (mmHg*min)	−178.54	173.93	−316.15	175.41	↘ 0.56
Rise time efficiency (min)	2.03	1.02	4.25	4.02	↓ 0.48
Median infusion (mL/min)	15.44	29.06	25.13	39.26	↘ 0.61
Mean infusion (mL/min)	29.56	22.92	51.67	27.11	↘ 0.57
Variable infusion rate (mL/min)	22.50	13.06	24.99	10.02	↘ 0.90

Results are shown as average and standard deviation values. Ratios between the first resuscitation and second resuscitation are shown to highlight large changes between both events. Arrows indicate when the ratio is greater or less than 1 to indicate which direction the performance metric differed between the resuscitation events.

**FIGURE 10:**
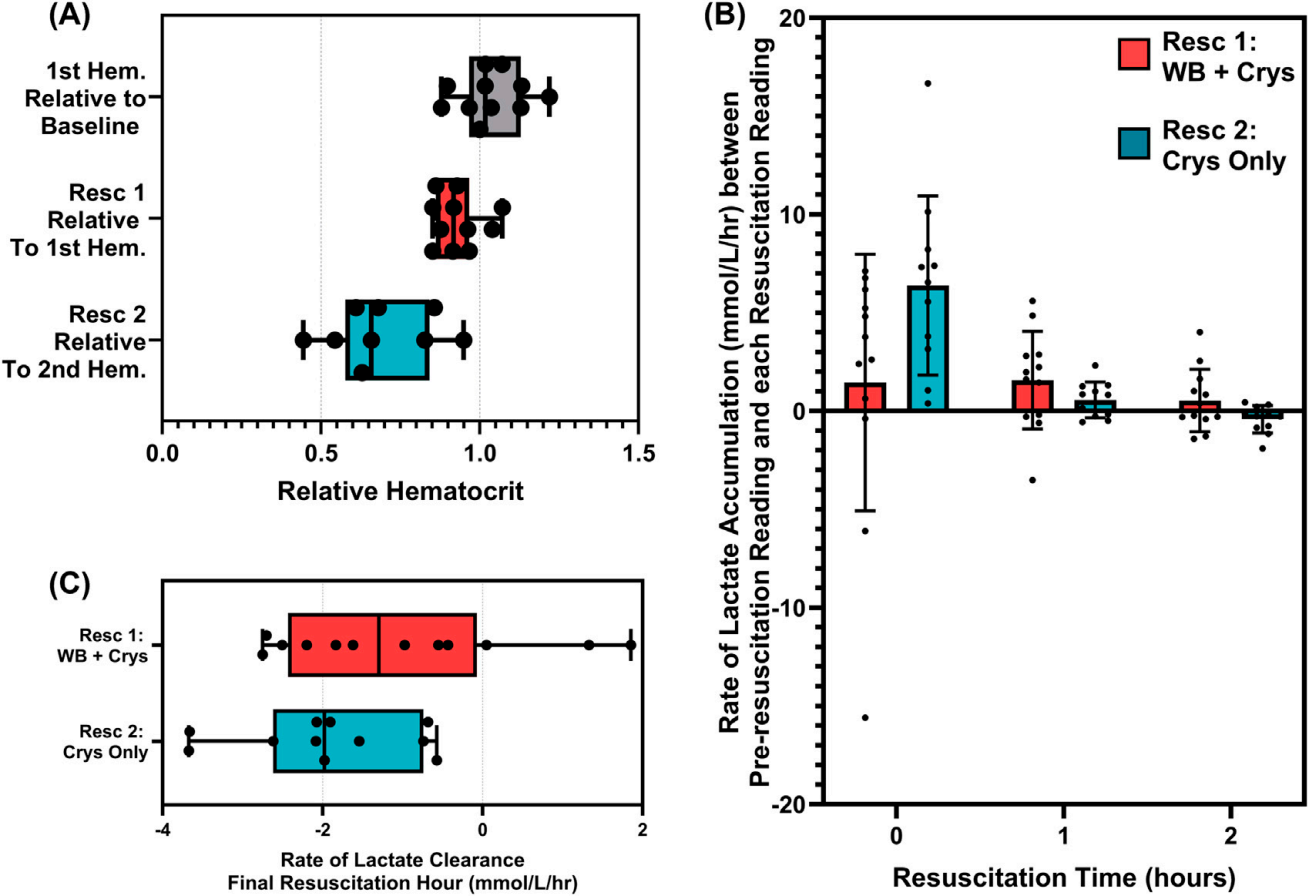
Comparison of hematocrit and lactate clearance trends for the first resuscitation (n = 12) and second resuscitation (n = 11) events. **(A)** Comparison of changes in hematocrit between baseline and hemorrhage, after the first resuscitation and after the second resuscitation. **(B)** Rate of lactate clearance based on the slope between the final blood lactate reading prior to resuscitation and after 0-, 1-, and 2-h resuscitation for both resuscitation events. **(C)** Comparison of the rate of blood lactate clearance during the final 1 h of each resuscitation event.

Lastly, we evaluated both resuscitation events for differences in hematocrit and lactate clearance. For hematocrit, there were limited effects of the first resuscitation, with levels only reducing to 93% of pre-resuscitation readings (Figure 10A). However, the second resuscitation event resulted in hematocrit decreasing to 69%, likely due to the dilution effect of a much higher volume of the infused crystalloid. For lactate clearance, there was a continued increase in lactate immediately after reaching the target MAP due to lactate clearance being a lagging indicator of hemorrhagic shock (Figure 10B). However, the crystalloid-only infusion accelerated lactate clearance, and during the final hour of the maintenance period, clearance rates were as high as 2 mmol/L/hr, while WB-based resuscitation only achieved 1.0 mmol/L/hr (Figure 10C).

## Discussion

Hemorrhage remains the leading cause of preventable death on the battlefield, but mortality can be reduced through hemorrhage control, followed by resuscitation to a physiologically normal blood pressure. Closed-loop control systems show potential to improve hemorrhagic shock patient outcomes by providing automated infusion control for resuscitation and maintenance at a target pressure for sustained durations. In this effort, we evaluated the performance of the ARC for providing individualized hemorrhagic shock resuscitation fluid therapy.

Overall, the ARC was successful in providing fluid infusion during the first resuscitation event of the study, where whole blood was used to reach the target MAP, followed by 120 min of successful maintenance of the target pressure with crystalloid solution. During the maintenance period, the ARC system kept the median absolute performance error below 5% of the target MAP, and overshoot was, on average, 14% of the target MAP. Although this may be high for traditional control systems, it is worth noting that, first, the ARC cannot decrease the pressure once the target pressure is surpassed as it can only deliver fluid, and second, pressure overshoot is much less of a concern during trauma resuscitation. If ARC performance was not penalized for overshooting, average resuscitation effectiveness was an impressive 93%. One cause of overshoot was the rise time efficiency of the controller to reach the target MAP, which, on average, was only 2 min when using whole blood. This rapid recovery rate was by design based on the initial testing of the ARC using a hardware-in-loop test platform, where its performance was evaluated across a wide range of hemorrhage situations (Snider et al., 2022a).

Based on aggregate metric scores, higher overall performance was measured when more rapid infusion paces were used with the ARC, but this rate can be adjusted as needed for *in vivo* medical applications. One key question remains for PCLCs for hemorrhage resuscitation, which makes tuning their design parameters more challenging: is it ideal to rapidly approach the target blood pressure at the expense of lower stability and possible target overshoot, or would it be preferred to have a more stable controller design at the expense of expediency in reaching the target blood pressure and possibly require more fluid to reach the target? This question relates to the safety of PCLCs more broadly, an issue that is likely to be solved at least in part by adding more capabilities to closed-loop control systems that would allow them to function more autonomously, such as by using population-informed particle filters (Tivay and Hahn, 2023), extended Kalman filters (Yin et al., 2022), and control barrier functions (Yin et al., 2023), resulting in safer and more effective control systems. Whatever the solution, it is clear that a great deal more *in vivo* testing is needed to answer this question. Nevertheless, this study still proves the potential utility of the ARC for providing fluid infusion at or near the point of injury to offload medical providers’ cognitive burden from this task.

The ARC was further evaluated during a second resuscitation event, where only crystalloid was used as the infusate to reach and maintain the target pressure. In doing so, there were notable physiological and performance metric differences between the two resuscitation events. Overall, the resuscitation was slower in reaching the target MAP with crystalloid, and higher infusion rates were required to maintain the pressure at the target MAP throughout the hold period. Conversely, the MAP signal was more stable, resulting in reduced wobble and less target overshoot. This combination of performance metrics makes sense from our previous characterization of differences between WB and crystalloid pressure–volume dynamics (Berard et al., 2022). Although WB resuscitation remained linear across all MAP values, crystalloid-based resuscitation began to lose volume responsiveness as blood pressure increased to the point of reaching an equilibrium MAP. This is likely due to the increased redistribution of fluid to the interstitial space at higher pressures. A number of subjects during the second resuscitation were unable to reach the default target MAP without excessive fluid resuscitation. This raises the question as to whether it is preferred to lower the target MAP based on the subject’s hemodynamics or if it is more critical to reach a higher target MAP? The former approach would require a means of tracking fluid non-responsiveness and lowering the target MAP based on that metric. In this study, we utilized CVP data trends for assessing over-resuscitation as the crystalloid resulted in the CVP increasing rapidly, which may be a useful datum to consider when setting personalized target MAPs. If reaching a heightened target MAP is critical, the ARC would likely require the introduction of a vasopressor component in future iterations of the controller design.

The animal model we developed for this protocol benefited from an AutoBleed decision table for maintaining a hypovolemic deficit, which inadvertently resulted in some additional findings in this study. AutoBleed was successful in resisting the animals’ natural physiological compensatory mechanisms, allowing for a sustained shock injury and further confining the changes in the MAP to the influence of the ARC alone. The performance error always trended in the positive direction as AutoBleed more often had to continue hemorrhaging the subject, as opposed to providing fluid to increase the MAP. AutoBleed could only perform this job with some filters to disregard MAP noise due to blood samples being taken for blood lactate throughout its use.

Due to nationwide ketamine shortages throughout this effort, the animal study became nearly equally split between ketamine as the primary anesthetic agent and isoflurane. The time to reach a lactate target of 4 mmol/L was significantly less when using ketamine, and, as such, the rate of blood lactate increase was higher, i.e., nearly two times as quick as when using isoflurane. This could hint at a hemorrhagic shock-protective effect offered by isoflurane, as has been shown in rat studies (Shi et al., 2018; Dai et al., 2020). However, this still requires further exploration. It also highlights the importance of adding anesthetic agents as a user input to the ARC as the overall hemodynamics may be impacted by this decision. Future ARC iterations could have different pre-programmed hemodynamic logic based on the anesthetic used.

The primary limitation for the ARC at this point is that its only goal is to provide fluid to reach a target MAP, which oversimplifies some of the complexities associated with hemorrhage resuscitation. Additional business rules are needed to inform the end user of the patient status, as well as to manage the ARC’s control logic under certain conditions. For instance, crystalloid-only resuscitation sometimes reached a point where the animal was not responsive to additional fluid infusion even though target MAP had not been achieved yet. In those situations, in its current form, the ARC will continue administering fluid, likely resulting in over-resuscitation, as well as a number of severe associated complications such as cardiac failure and pulmonary edema. As we mentioned, one of the subjects underwent cardiac failure during high fluid infusion rates, as suggested by the ARC during crystalloid infusion. Business rules can help provide some improved logic in these situations, allowing the end user to either adjust the target MAP to a lower value or provide vasopressor therapy to overcome these challenges. Another ARC management aspect to integrate is the knowledge of ongoing anesthesia decisions. Oftentimes, as anesthesia infusion rates were manually adjusted, the subjects’ volume responsiveness and MAP reacted accordingly. It would be ideal to analyze whether physiological changes are due to the subjects’ condition or anesthesia, which may improve ARC performance in the future.

There are additional limitations to this study and the ARC beyond the housekeeping rules. First, the two hemorrhage events used in this study are not independent. Compounding physiological changes measured during the second resuscitation are not necessarily due to crystalloid infusion alone as the multi-trauma faced by the subject provides a major confounding factor. The primary goal of this study was to confirm the ARC’s performance with WB, so randomizing the order of the fluid therapies was not done in this study. However, order randomization could be performed in future iterations of this effort to better understand the difference in performance due to the fluid type. Second, the WB resuscitation therapy used an arbitrary set point of switching to crystalloid after 10 min at the target MAP. There is no clear physiological basis for making this switch currently, and it would be advantageous if the ARC can evaluate physiological stability and modify its fluid infusate based on that logic. For instance, there were delayed changes in mixed venous saturation, cardiac output, and stroke volume variability that could be tracked in real time by further ARC iterations for guiding infusate swap-over decisions. Lastly, the ARC in its current form relies on an invasive arterial signal as input for fluid infusion rate calculations. Although this may be acceptable in some situations, in a pre-hospital setting, and especially during combat casualty care, invasive arterial lines may not be easily placed for driving fluid management.

## Conclusion

Hemorrhagic shock and the associated mortality can be improved if proper fluid therapy is provided in a timely manner, especially during combat casualty care where definitive care may not be available for extended periods of time. The ARC technology evaluated here in a large animal hemorrhagic shock study allowed for rapid resuscitation to a target MAP and effectively maintaining the pressure indefinitely using multiple infusate types. The next steps in this effort will integrate physiological logic rules to better mimic clinical fluid resuscitation treatment, such as rules for when vasopressor treatment may be needed to improve fluid therapy. Another next step for this technology will include the modification of the ARC to support different sensor inputs such as non-invasive pressure inputs or other metrics that could be used to drive hemorrhage resuscitation more easily. In summary, the ARC was proven to be an effective physiological closed-loop controller at driving resuscitation in a hemorrhagic shock live animal study, a critical next step in developing autonomous hemorrhage resuscitation in the prehospital setting.

## Data Availability

The datasets presented in this article are not readily available because they have been collected and maintained in a government-controlled database that is located at the US Army Institute of Surgical Research. As such, these data can be made available through the development of a Cooperative Research and Development Agreement (CRADA) with the corresponding author. Requests to access the datasets should be directed to Eric Snider, eric.j.snider3.civ@health.mil.
