# Supplementary material for: In vivo evaluation of an adaptive resuscitation controller using whole blood and crystalloid infusates for hemorrhagic shock
Source: Front Bioeng Biotechnol. 2024 Nov 8;12:1420330. doi: 10.3389/fbioe.2024.1420330 (PMC11581866; doi:10.3389/fbioe.2024.1420330)
Supplement: Supplementary file 1 [file DataSheet1.docx]

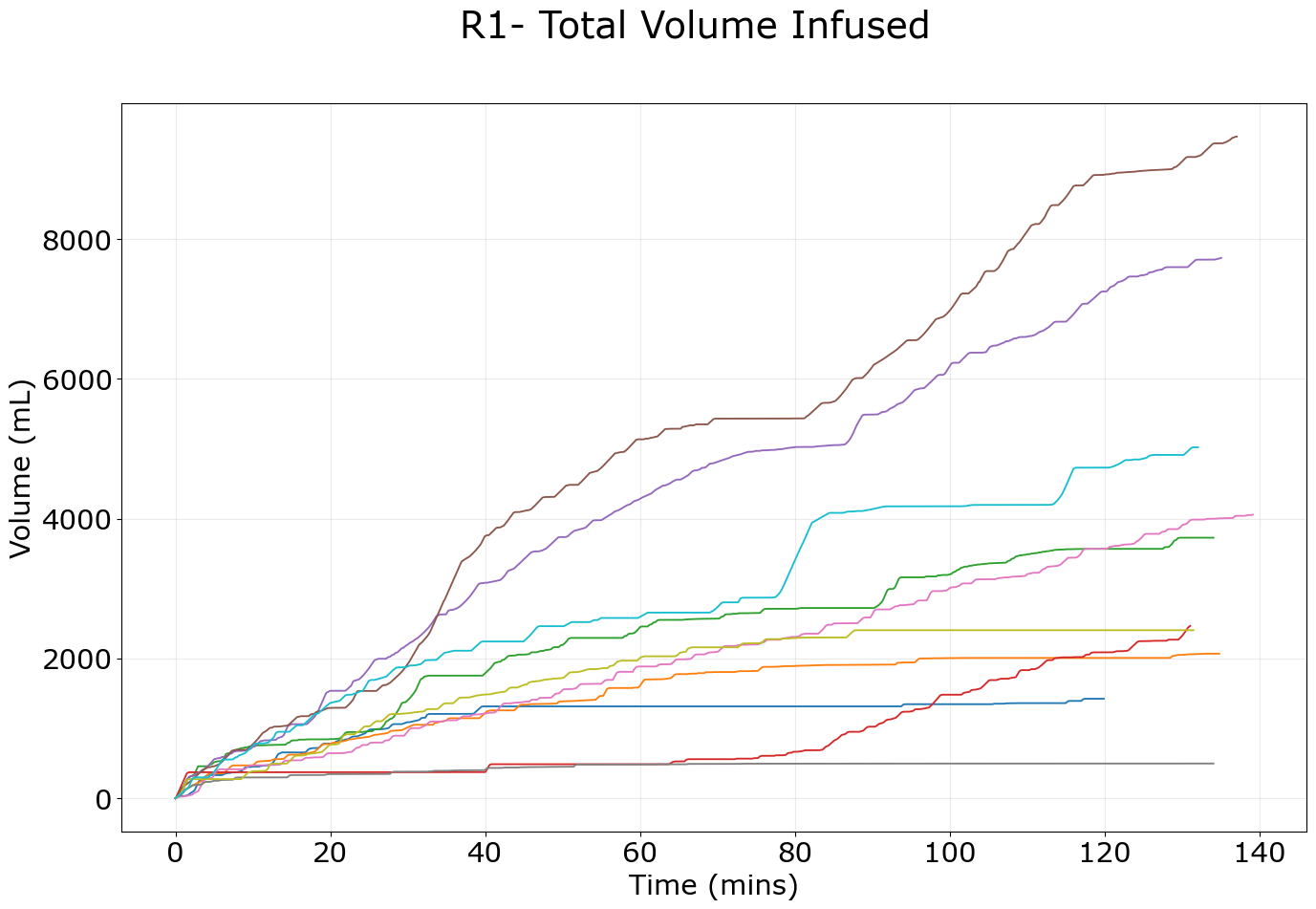
**Supplementary Figure 1**. Total infused volume vs. time during the first resuscitation event for each subject who completed this phase of the study.


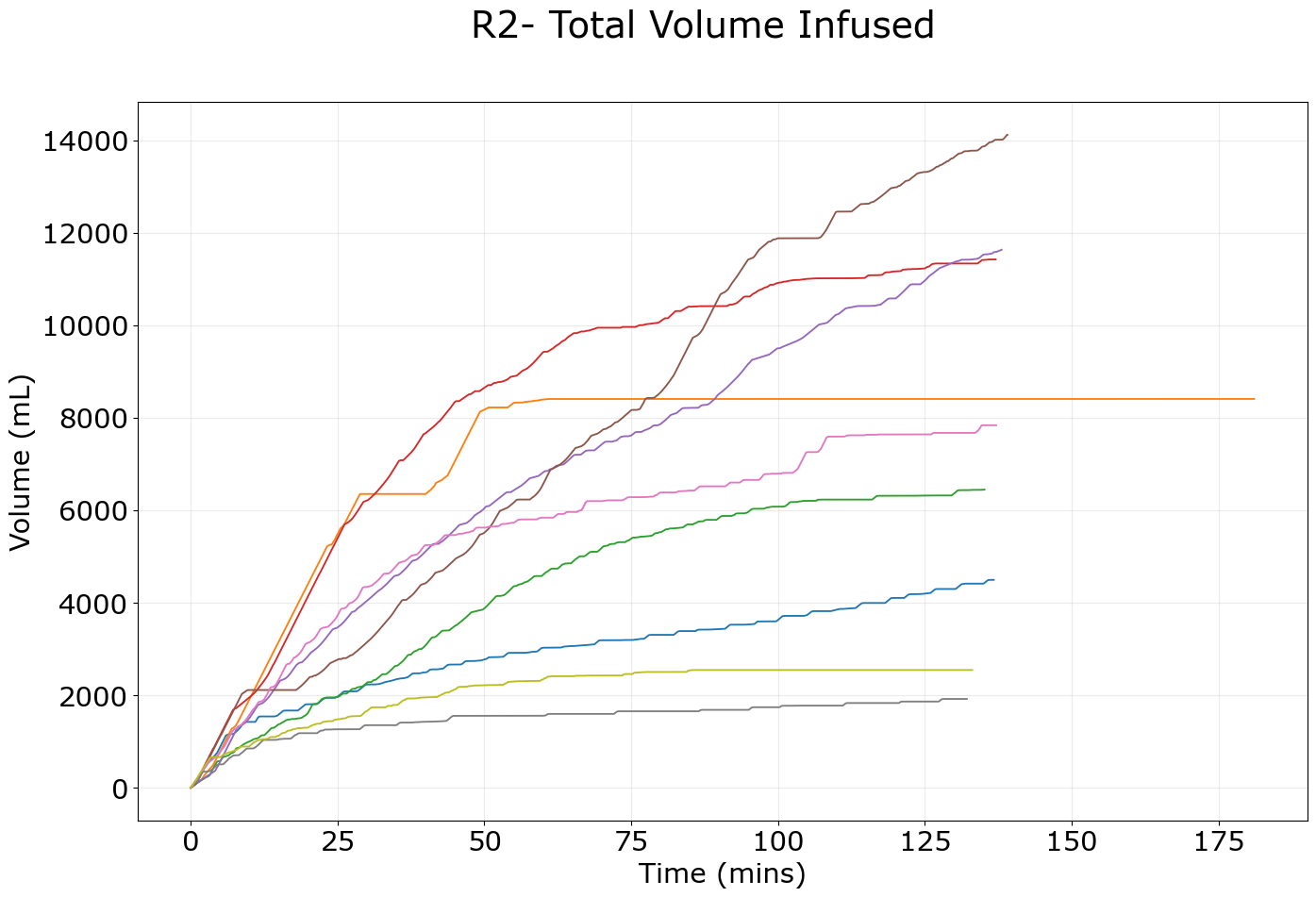


**Supplementary Figure 2.** Total infused volume vs. time during the second resuscitation event for each subject who completed this phase of the study. Note: one subject had catheter fluid flow issues that required restarting the controller during the experimental phase, resulting in the total duration of this phase being much longer for one subject.
